# A Concise and Geometrically Exact Planar Beam Model for Arbitrarily Large Elastic Deformation Dynamics

**DOI:** 10.3389/frobt.2020.609478

**Published:** 2021-05-25

**Authors:** Gerold Huber, Dirk Wollherr, Martin Buss

**Affiliations:** Chair of Automatic Control Engineering (LSR), Department of Electrical and Computer Engineering, Technical University of Munich (TUM), Munich, Germany

**Keywords:** Kirchhoff–Love beam, elastic deformation, large deformation, planar deflection, geometrically exact, PDE, FEM

## Abstract

The potential of large elastic deformations in control applications, e.g., robotic manipulation, is not yet fully exploited, especially in dynamic contexts. Mainly because essential geometrically exact continuum models are necessary to express these arbitrarily large deformation dynamics, they typically result in a set of nonlinear, coupled, partial differential equations that are unsuited for control applications. Due to this lack of appropriate models, current approaches that try to exploit elastic properties are limited to either small deflection assumptions or quasistatic considerations only. To promote further exploration of this exciting research field of large elastic deflection control, we propose a geometrically exact, but yet concise a beam model for a planar, shear-, and torsion-free case without elongation. The model is derived by reducing the general geometrically exact the 3D Simo–Reissner beam model to this special case, where the assumption of inextensibility allows expressing the couple of planar Cartesian parameters in terms of the curve tangent angle of the beam center line alone. We further elaborate on how the necessary coupling between position-related boundary conditions (i.e., clamped and hinged ends) and the tangent angle parametrization of the beam model can be incorporated in a finite element method formulation and verify all derived expressions by comparison to analytic initial value solutions and an energy analysis of a dynamic simulation result. The presented beam model opens the possibility of designing online feedback control structures for accessing the full potential that elasticity in planar beam dynamics has to offer.

## 1 Introduction

Dynamic robotic manipulation of highly deformable objects is still a rarely considered field in literature due to a lack of appropriate dynamic models. Elastic objects in robotic manipulation are usually either considered only quasistatically (Bretl and McCarthy, 2014) or dynamically under the assumption of small deflections which in return results in applications where elasticity is often treated as an undesired property that needs to be avoided or compensated (Tavasoli, 2015). There are some approaches that suggest exploiting elasticity instead, e.g., in terms of safety (Bicchi and Tonietti, 2004; Haddadin et al., 2009), payload estimation (Malzahn et al., 2015), or energy storage (Tantanawat and Kota, 2007; Haddadin et al., 2011). Some authors exploit elasticity also for quasistatic manipulation (McCarragher, 2000) or even to design new strategies for dynamic manipulation, e.g., Pekarovskiy et al. (2014) or Pham and Pham (2017). Nonetheless, we propose that elastic dynamics have even more potential that can be exploited when large deflections are considered.

Models that allow taking advantage of such arbitrarily large deformations need to fulfill two main criteria. They have to

1. describe the dynamics *geometrically exact*, i.e., independently of the magnitude of deformation
2. be concise and simple enough to allow the design of online feedback control

However, such models are not yet available in literature. In this study, we start filling this gap for the case of a planar elastic beam undergoing pure bending, by proposing a single-dimensional dynamic continuum model.

### 1.1 Related Work

Large deflection dynamics are typically treated with one of two approaches—discretized approximations using a finite element method (FEM) or continuum models expressed as partial differential equations (PDEs). The FEM-based descriptions, for which recent literature such as the study of Duriez (2013) reaches real-time capable control, are limited to quasistatic deformable structures, as the computational cost of FEM descriptions for true dynamic large deformation online feedback control is still out of reach. Whereas, the geometrically exact continuum models result in multivariate and highly coupled nonlinear PDE systems, and control approaches are thus limited to oscillation damping (Ito, 2001; Hegarty and Taylor, 2012). Unlike existing control methods for linear beam models (Krstic et al., 2006a; Krstic et al., 2006b), the literature body on theory of nonlinear PDE systems is still too limited in its applicability for the complex expressions arising in these mechanical system models (Padhi and Ali, 2009). From a control point of view, however, it is well known that continuum models, unlike discretized approximations, do not face so called spillover phenomena that can lead to instabilities due to unmodeled high frequency dynamics (Meirovitch and Baruh, 1983). For these reasons, as a first contribution toward filling the discussed gap in literature, this work proposes a geometrically exact model of planar Euler–Bernoulli beam dynamics for arbitrarily large deflections that admit a surprisingly concise PDE formulation.

Although there are special purpose models for large deflection models, such as bullwhip dynamics by Bernstein et al. (1958) and McMillen and Goriely (2003) or dynamics of a fly fishing line by Spolek (1986), we are interested in more general beam dynamics that admit rope and cable dynamics as a special case of very low elastic stiffness. A vast literature body exists on 1D analytic beam theories alone. Therefore, because of the considerable computational advantage of 1D beam theories over 3D continuum mechanics theories, the latter will not be considered in this concise literature review. Starting with the first mathematical treatment of static elasticity by Galileo already in 1638, Hooke’s treatise of linear elasticity is followed in 1678. The first precise definition of the elastica (a thin strip of elastic material) problem was carried out by Jakob Bernoulli (1691), and he published its first solution in Bernoulli (1694). His nephew Daniel Bernoulli (1742) did not himself solve the problem, but he suggested Euler the use of variational analysis, who delivers a closed-form solution of the elastica in the study by Euler (1744). A more detailed and insightful mathematical historical overview of the elastica can be found in the study by Levien (2008). While Euler’s early work already predicts slender beam deformations with astounding precision, many authors built on this work to include further effects to account for more general conditions as well as geometries. It is said that Rayleigh (1877) added rotary inertia effects, and Timoshenko (1921) further enhanced the theory to account for shear effects. However, Elishakoff (2020) discusses original authors and naming of linear beam theories. He, e.g., mentioned that Bresse (1859) already included rotary inertia effects before Rayleigh, though the works were developed independently. Furthermore, a beam theory including shear effects was originally published in Timoshenko (1916), an earlier book in Russian language, where Timoshenko mentions to have developed the theory together with P. Ehrenfest. Elishakoff, therefore, suggests the historically justifiable name *Bresse–Rayleigh–Timoshenko–Ehrenfest beam theory*.

For large deflections, also referred to as *finite strain*, geometrically nonlinear models are necessary. Kirchhoff (1859) is a spatial generalization of the Euler–Bernoulli beam and allows modeling of 3D deformations through bending and torsion. The theory was later extended by Love (1892) to further account for axial tensions and is referred to as *Kirchhoff–Love beam theory*. Reissner (1972) added further measures to Kirchhoff’s theory, accounting for shear deformations in planar curves and later for space curves in Reissner (1981). Simo (1985) enhanced Reissner’s work in terms of approximations to what is nowadays known as *Simo–Reissner beam theory* or *geometrically exact beam theory*. To complete the overview, it is also worth mentioning that reduced versions of these two well-known theories have been proposed that neglect torsion modes, e.g., the study by Meier et al. (2015) for the Kirchhoff–Love and Romero et al. (2014) for the Simo–Reissner case. Meier et al. (2019) gave a more in-depth overview and analysis of these nonlinear beam theories. Table 1 shows how our proposed model fills the current literature gap of a concise model for geometrically exact descriptions.

**TABLE 1:** Comparison of this work to the most commonly used beam theories. While the proposed model is limited to planar bending, it offers a geometrically exact description in a single-dimensional equation.

	Beam model	PDEs	LateralBending	RotaryInertia	ShearDeformation	AxialTorsion	AxialTension
Geometrically exact	Simo–Reissner	Six	Spatial	✓	✓	(✓)	✓
	Kirchhoff–Love	Four	Spatial	✗	✗	(✓)	✓
	Kirchhoff	Four	Spatial	✗	✗	(✓)	✗
		Proposed model	One	Planar	✗	✗	✗	✗
Linearized	Timoshenko	Two	Planar	✓	✓	✗	✗
	Rayleigh	One	Planar	✓	✗	✗	✗
	Euler–Bernoulli	One	Planar	✗	✗	✗	✗

### 1.2 Contribution

The main contribution of this work is twofold. We provide

• the first single-dimensional, geometrically exact, dynamic beam model, and
• a method for incorporating boundary conditions in a FEM formulation, for cases where the FEM model has only descriptive variables of higher-order derivatives than the boundary condition itself

### 1.3 Outline

In Section 2, we derive the model via step-by-step reduction, starting from the general Simo–Reissner beam theory, extracting first the Kirchhoff–Love beam theory, followed by special case assumptions — isotropic, torsion-free, inextensible, and planar bending. The translation into a FEM description is explained in Section 3, including our proposal for incorporating position-level boundary conditions into the tangent angle PDE system. This FEM description is applied for the simulation verification in Section 4. The work is concluded in the final Section 5.

## 2 Modeling

This section outlines the model derivation, starting from a general 3D theory. After reducing the model by gradually introducing assumptions, we couple the Cartesian coordinates to achieve an expression in a single PDE.

### 2.1 Nomenclature

This work follows the convention of using lowercase bold variables for vectors and uppercase bold variables for matrices. All nonbold variables are scalars. Furthermore, subscript annotations are reserved for index notation of multidimensional variables as well as expressing partial derivatives, whereas superscript annotations are part of the variable specification. Also, note that we omit explicit listing of function parameters whenever it is clear from the context to not unnecessarily clutter the notation. A list of the most frequently used variables at the end of this work is given.

### 2.2 Model Reduction

The presented geometrically exact model for a planar Euler–Bernoulli beam is found via reduction of the general 3D Simo–Reissner beam theory. We gradually introduce further assumptions to simplify the dynamic governing equations of the beam. Please note that we only define the individual components of the equations once necessary to keep the derivation clear and easy to follow. The resulting model forms a special case of a planar Kirchhoff–Love beam theory parametrized solely in the curve tangent angle, in an analog to Euler’s elastica (Euler, 1744). A more in-detail analysis and discussion of the general Simo–Reissner and Kirchhoff–Love beam theories, including the special cases of isotropic cross-sections and torsion-free formulations, can be found in Meier et al. (2019).

#### General Simo–Reissner Beam Theory

The first theory that accounts for very general 3D beam deformations including spatial bending, torsion, axial tension, and shear deformation was published by Simo (1985). The consideration of shear effects makes it an adequate theory for thick rod dynamics. It is also been denoted by *geometrically exact beam theory* because the description is consistent at the deformed state regardless of the magnitude of displacements, rotations, and strains, cf. Crisfield and Jelenić (1999). Simo himself also used the term *finite strain beam formulation*.

The strong form of the Simo–Reissner beam theory, cf. Simo (1985), is a system of six coupled PDEs and can be stated with the equilibrium equations

$${\left(f^{\text{internal}}\right)}_{l}+f^{\text{ext}}+f^{\text{inertia}}=0,$$ (1a)

$${\left(m^{\text{internal}}\right)}_{l}+{\left(r\right)}_{l}\times f^{\text{internal}}+m^{\text{ext}}+m^{\text{inertia}}=0,$$ (1b)

where the internal force $f^{\text{internal}}$ and moment vector $f^{\text{internal}}$ results form internal stresses acting on the beam cross-section area at point ***r*** of the beam center line. The quantities $f^{\text{ext}}$ and $m^{\text{ext}}$ account for externally imposed forces, and $f^{\text{intertia}}$ and $m^{\text{intertia}}$ are the components due to inertia effects. The detailed constitutive equations that relate $f^{\text{internal}}$ and $m^{\text{internal}}$ to the first Piola–Kirchhoff stress tensor require an introduction into 3D continuum mechanics and is omitted in this work. Instead, we point the interested reader to related text books such as by Gurtin (1982) and define the expressions only after reduction to the specified special cases.

In the following, all objective deformation measures are chosen to be work conjugated to the material stress resultants in Eq. 1. We further assume a hyperelastic constitutive relation between these kinetic and kinematic quantities.

#### Assumption: Vanishing Shear Strains (Kirchhoff–Love Beam Theory)

**FIGURE 1:**
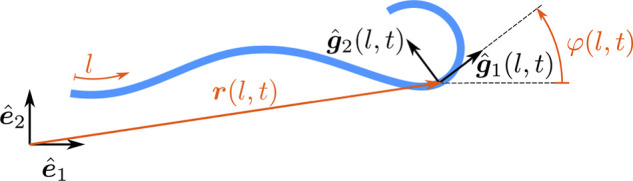
Illustration of the used variables to describe beam deformation.

Neglecting shear deformations and assuming that the cross-section is always perpendicular to the center line of the beam, the change in internal forces ${\left(f^{\text{internal}}\right)}_{l}$ can be split up into a parallel component $\|{\left(f^{\text{internal}}\right)}_{l}$ and (finternal)⊥l, a component perpendicular to the center line. Furthermore, the moment balance Eq. 1b reduces to the projection onto the center line tangential base vector ${\hat{g}}_{1}$, Figure 1 for an illustration. The Kirchhoff–Love beam equations are thus given with

‖(finternal)l+((r)l‖(r)l‖22×((minternal)l+mext+minertia))l︸(finternal)⊥l+fext+finertia=0, (2a)

$${\hat{g}}_{1}^{T}\left({\left(m^{\text{internal}}\right)}_{l}+m^{\text{ext}}+m^{\text{inertia}}\right)=0,$$ (2b)

where Eq. 2b is now a scalar expression, and the beam model is thus reduced to 4 PDEs.

#### Assumption: Initially Straight and Isotropic

If now an initially straight beam with an isotropic cross-section is assumed for the hyperelastic beam, the components of Eq. 2 are given with

$$\|{\left(f^{\text{internal}}\right)}_{l}+{\left(\frac{{\left(r\right)}_{l}}{{\left\|{\left(r\right)}_{l}\right\|}_{2}^{2}}\times \left(\underset{{\left(m^{\mathrm{int}ernal}\right)}_{l}}{\underset{︸}{EI{\left(\kappa \right)}_{l}}}+m^{\text{ext}}+m^{\text{inertia}}\right)\right)}_{l}+f^{\text{ext}}\underset{f^{\text{inertia}}}{\underset{︸}{-\rho A{\left(r\right)}_{tt}}}=0$$ (3a)

$$2GI{\left(\kappa_{1}\right)}_{l}+m_{1}^{\text{ext}}-2\rho I{\left(\omega_{1}\right)}_{t}=0,$$ (3b)

with the Young modulus *E*, inertia *I*, density *ρ*, cross-section area *A,* and the curvature vector

$$\kappa :=\frac{{\left(r\right)}_{l}\times {\left(r\right)}_{ll}}{{\left\|{\left(r\right)}_{l}\right\|}_{2}^{2}}.$$ (4)

The components $\kappa_{1}$, $m_{1}^{\text{ext}}$, and $\omega_{1}$ in Eq. 3b relate to the curvature, externally imposed moment, and angular velocity along the tangential direction of the beam.

#### Assumption: Torsion-Free

Assuming pure bending and no torsional effects, the inertia moment and the moment balance Eq. 3b vanish completely. Moreover, only the perpendicular component of the external moment affects the force balance equation

(EAϵg^1)l︸‖(finternal)l+((r)l‖(r)l‖22×(EI(κ)l+mext⊥))l+fext−ρA(r)tt=0, (5)

with the axial tension parameter $\varepsilon :={\left\|{\left(r\right)}_{l}\right\|}_{2}-1$, which considers that the relation

$${\left\|\frac{\partial r^{\text{undef}}}{\partial l}\right\|}_{2}=1,$$ (6)

of the undeformed beam, does not in general hold for the deformed case

$${\left\|\frac{\partial r}{\partial l}\right\|}_{2}\neq 1,$$ (7)

due to possible elongations in the beam structure.

#### Assumption: Inextensible Beam

If it is assumed that the beam does not undergo axial elongations, the gradient Eq. 7 does always equals 1. Hence, the axial tension parameter *ϵ* evaluates to

$$\varepsilon \left(l\right)\equiv \;{\left\|\frac{\partial r}{\partial l}\right\|}_{2}-1\equiv 0,$$ (8a)

and thus, ^∥^ ${\left(f^{\text{internal}}\right)}_{l}$ vanishes. The beam model Eq. 5 consequently further simplifies to

((r)l×(EI((r)l×(r)ll)l+mext⊥))l+fext−ρA(r)tt=0. (8b)

However, unlike the previous assumptions that can be incorporated implicitly with an adequate choice of parametrization variables, it is in general difficult to find such a set of variables that fulfill the inextensibility constraint Eq. 8a by construction. A common practice to enforce the equality constraint Eq. 8a on the simulation result in a weak sense, i.e., in an integral form instead of point-wise, is by means of extending the weak form of the model Eq. 8b with a Lagrange multiplier potential, cf. Meier et al. (2019).

The following assumption of pure planar bending, however, does again permit a parametrization that fulfills this constraint directly in the strong sense, i.e., for every point along the beam.

#### Assumption: Pure Planar Bending

The last step in the model reduction is the restriction to pure planar bending. For the remainder of this section, we will switch to a component-wise notation in Cartesian coordinates. The beam model from Eq. 8b reduces to two coupled PDEs and is fully described by

$${\left[\begin{array}{l} {\left(y\right)}_{l}\left(EI{\left({\left(x\right)}_{l}{\left(y\right)}_{ll}-{\left(y\right)}_{l}{\left(x\right)}_{ll}\right)}_{l}{+}^{⊥}m_{z}^{\text{ext}}\right)\\ -{\left(x\right)}_{l}\left(EI{\left({\left(x\right)}_{l}{\left(y\right)}_{ll}-{\left(y\right)}_{l}{\left(x\right)}_{ll}\right)}_{l}{+}^{⊥}m_{z}^{ext}\right) \end{array}\right]}_{l}+\left[\begin{array}{l} f_{x}^{\text{ext}}\\ f_{y}^{\text{ext}} \end{array}\right]-\rho A{\left[\begin{array}{l} x\\ y \end{array}\right]}_{tt}=0,$$ (9a)

and the additional inextensibility constraint

$${\left\|\left[\begin{array}{l} {\left(x\right)}_{l}\\ {\left(y\right)}_{l} \end{array}\right]\right\|}_{2}-1\equiv 0$$ (9b)

because the third row of the vector equation Eq. 8b vanishes for the purely planar problem. Thus, the remaining external inputs are forces $f^{\text{ext}}$ in the *xy*-plane, as well as the external moment mzext⊥ is perpendicular to this plane.

With the curve tangent angle φ defined as the angle between the tangent vector of the deformed beam center line ${\hat{g}}_{1}$ and its undeformed counterpart ${\hat{e}}_{1}$, the beam curvature Eq. 4 can be expressed as

$$\kappa^{\text{planar}}:=\left[\begin{array}{c} 0\\ 0\\ {\left(\varphi \right)}_{l} \end{array}\right],$$ (10)

for the shear-free, planar, inextensible case. Furthermore, it allows stating the geometric identities

$${\left(x\right)}_{l}=\cos\left(\varphi \right),$$ (11a)

$${\left(y\right)}_{l}\equiv \sin\left(\varphi \right).$$ (11b)

The planar beam model Eq. 9 can thus be stated as

[sin(φ)(EI(φ)ll+mzext⊥)−cos(φ)(EI(φ)ll+mzext⊥)]l+[fxextfyext]−ρA[xy]tt=0, (12)

a beam model in a mixed form containing Cartesian coordinates as well as the curve tangent angle as describing variables. In the remainder of this Section, Eq. 12 is the starting point to first develop the static case followed by the general dynamic case, both entirely expressed in the curve tangent angle.

#### 2.2.1 Static Beam Model Expressed in the Curve Tangent Angle

Only considering solutions in a static equilibrium, the Cartesian acceleration terms in Eq. 12 vanish and

[sin(φ)(EI(φ)ll+mzext⊥)−cos(φ)(EI(φ)ll+mzext⊥)]l+[fxextfyext]=0 (13)

remains. By computing all derivatives,

[cos(φ)(φ)l(EI(φ)ll+mzext⊥)+sin(φ)(EI(φ)lll+(mzext⊥)l)sin(φ)(φ)l(EI(φ)ll+mzext⊥)−cos(φ)(EI(φ)lll+(mzext⊥)l)]+[fxextfyext]=0, (14)

and rotating the equations from their Cartesian xy coordinate system by *φ* around the *z*-axis, by premultiplying Eq. 14 with the rotation matrix

$$R_{z}\left(\varphi \right):=\left[\begin{array}{cc} \cos\left(\varphi \right) & \sin\left(\varphi \right)\\ -\sin\left(\varphi \right) & \cos\left(\varphi \right) \end{array}\right],$$ (15)

which allows extracting the components perpendicular ⊥ and parallel ∥ to the beam center line

[(φ)l(EI(φ)ll+mzext⊥)−(φ)l(EI(φ)lll+(mzext⊥)l)]+[fext∥fext⊥]=0. (16)

The fact that no Cartesian xy parameter of the beam description remains but is rather described in the curve tangent angle cta alone means that the geometric identities Eq. 11 and the planar inextensibility constraint Eq. 9b are now implicitly fulfilled by construction. No further treatment such as Lagrangian multipliers are thus necessary, in contrast to the above beam models (Eq. 8b and Eq. 9). In case of an absent external force $f^{\text{ext}}$, the static beam model Eq. 16 even admits a simple analytic solution. If a nontrivial curvature ${\left(\varphi \right)}_{l}\neq 0$ is assumed, Eq. 16 reduces to

(φ)ll=−1EImzext⊥, (17)

which can be integrated twice and yields a unique solution if boundary conditions are applied.

#### 2.2.2 Dynamic Beam Model Expressed in the Curve Tangent Angle

To also fully state the dynamic planar beam model in terms of the curve tangent angle, the Cartesian xy acceleration terms in Eq. 12 remain to be expressed in terms of the curve tangent angle *φ*. This is achieved by differentiating the system of equations Eq. 12 w.r.t. the beam parameter *l*. Assuming no buckling of the object, *x* and *y* have continuous derivatives, and thus, Schwarz’s theorem allows changing the order of the derivations. Applying the geometric identities (Eq. 11) now also to the acceleration terms, leads to

$${\left[\begin{array}{c} \sin\left(\varphi \right){\left(\phi \right)}_{ll}\\ -\cos\left(\varphi \right){\left(\phi \right)}_{ll} \end{array}\right]}_{ll}+{\left[\begin{array}{c} f_{x}^{\text{ext}}\\ f_{y}^{\text{ext}} \end{array}\right]}_{l}-\rho A{\left[\begin{array}{c} \cos\left(\varphi \right)\\ \sin\left(\varphi \right) \end{array}\right]}_{tt}=0,$$ (18a)

with the auxiliary variable

(ϕ)ll:=EI(φ)ll+mzext⊥, (18b)

a PDE system entirely expressed in terms of the curve tangent angle *φ*. As for the static case (Eq. 16), the geometric identities fulfill the inextensibility constraint Eq. 9b by construction; thus, no special consideration is necessary. Expanding all partial derivatives and grouping the trigonometric terms, Eq. 18a yields

$$\left[\begin{array}{c} \cos\left(\varphi \right)\left(2{\left(\varphi \right)}_{l}{\left(\phi \right)}_{lll}+\rho A{\left(\varphi \right)}_{t}^{2}\right)-\sin\left(\varphi \right)\left({\left(\varphi \right)}_{l}^{2}{\left(\phi \right)}_{ll}-{\left(\phi \right)}_{llll}-\rho A{\left(\varphi \right)}_{tt}\right)+{\left(f_{x}^{\text{ext}}\right)}_{l}\\ \sin\left(\varphi \right)\left(2{\left(\varphi \right)}_{l}{\left(\phi \right)}_{lll}+\rho A{\left(\varphi \right)}_{t}^{2}\right)+\cos\left(\varphi \right)\left({\left(\varphi \right)}_{l}^{2}{\left(\phi \right)}_{ll}-{\left(\phi \right)}_{llll}-\rho A{\left(\varphi \right)}_{tt}\right)+{\left(f_{x}^{\text{ext}}\right)}_{l} \end{array}\right]=0.$$ (19)

Similar to the static case, premultiplying the entire system with the rotation matrix $R_{z}\left(\varphi \right)$ from Eq. 15 again extracts the components parallel and perpendicular to the beam center line. The only acceleration term ${\left(\varphi \right)}_{tt}$, however, appears solely in the perpendicular direction

EI((φ)l2(φ)ll−(φ)llll)+((φ)l2mzext⊥−(mzext⊥)ll)+(fext⊥)l−ρA(φ)tt=0, (20)

which in the case of no external inputs admits the very concise strong form

$${\left(\varphi \right)}_{tt}=c\left({\left(\varphi \right)}_{l}^{2}{\left(\varphi \right)}_{ll}-{\left(\varphi \right)}_{llll}\right)\;\text{with}\;c:=\frac{EI}{\rho A},$$ (21)

as a single PDE governing the beam dynamics in a single parameter *φ*.

While this reduced model is relevant for PDE controller development, it is not directly applicable for use in simulations. We therefore present in the following section a respective approximation with a system of ordinary differential equations (ODEs), in terms of a FEM formulation.

## 3 FEM Formulation

In this section, we outline the development of a FEM simulation procedure, starting from the development of the weak form of the beam model (Eq. 21), without considering external forces. After transforming the integrodifferential weak form into a system of nonlinear ODEs of thee second order in time via a Bubnov–Galerkin approximation, a finite element discretization leads to a simulation procedure.

### 3.1 Weak Form of Large Deformation in the Curve Tangent Angle

The weak form of Eq. 21 is found by multiplying the equation with the test function δφ and integrate over the beam length l=[0,L]:

$$\frac{1}{c}\int_{0}^{L}{\left(\varphi \right)}_{tt}\delta \varphi \text{d}l=\int_{0}^{L}{\left(\varphi \right)}_{l}^{2}{\left(\varphi \right)}_{ll}\delta \varphi \text{d}l-\int_{0}^{L}{\left(\varphi \right)}_{llll}\delta \varphi \text{d}l.$$ (22)

A sequence of integrations by parts will lead to the final weak form. In the first intermediate step,

$$\int_{0}^{L}{\left(\varphi \right)}_{l}^{2}{\left(\varphi \right)}_{ll}\text{d}l={{\left(\varphi \right)}_{l}^{3}|}_{0}^{L}=\int_{0}^{L}2{\left(\varphi \right)}_{l}^{2}{\left(\varphi \right)}_{ll}\text{d}l$$

allows to solve the integral

$$\int_{0}^{L}{\left(\varphi \right)}_{l}^{2}{\left(\varphi \right)}_{ll}\text{d}l={\frac{1}{3}{\left(\varphi \right)}_{l}^{3}|}_{0}^{L}.$$

Using this result, the terms on the right-hand side of Eq. 22 result in

$$\int_{0}^{L}\left({\left(\varphi \right)}_{l}^{2}{\left(\varphi \right)}_{ll}\right)\delta \varphi \text{d}l={\frac{1}{3}{\left(\varphi \right)}_{l}^{3}\delta \varphi |}_{0}^{L}-\frac{1}{3}\int_{0}^{L}{\left(\varphi \right)}_{l}^{3}{\left(\delta \varphi \right)}_{l}\text{d}l$$ (23a)

and

$$\int_{0}^{L}{\left(\varphi \right)}_{llll}\delta \varphi \text{d}l={\left(\varphi \right)}_{lll}{\delta \varphi |}_{0}^{L}-\int_{0}^{L}{\left(\varphi \right)}_{lll}{\left(\delta \varphi \right)}_{l}\text{d}l={\left(\varphi \right)}_{lll}{\delta \varphi |}_{0}^{L}-{\left(\varphi \right)}_{ll}{{\left(\delta \varphi \right)}_{l}|}_{0}^{L}+\int_{0}^{L}{\left(\varphi \right)}_{ll}{\left(\delta \varphi \right)}_{ll}\text{d}l,$$ (23b)

which lead to the final dynamic equations in a weak form

$$\frac{1}{c}\int_{0}^{L}{\left(\varphi \right)}_{tt}\delta \varphi \text{d}l={\left[-{\left(\varphi \right)}_{lll}\delta \varphi +{\left(\varphi \right)}_{ll}{\left(\delta \varphi \right)}_{l}+\frac{1}{3}{\left(\varphi \right)}_{l}^{3}\delta \varphi \right]}_{0}^{L}-\int_{0}^{L}{\left(\varphi \right)}_{ll}{\left(\delta \varphi \right)}_{ll}+\frac{1}{3}{\left(\varphi \right)}_{l}^{3}{\left(\delta \varphi \right)}_{l}\text{d}l$$ (24)

that builds the basis for the following FEM formulation. The function *φ* as well as the variation δφ have to be the members of the Sobolev space ${ℋ}^{2}$, where

$${ℋ}^{k}:=\left\{w|w\in {ℒ}_{2},\frac{\partial w}{\partial x}\in {ℒ}_{2},\ldots ,\frac{\partial^{k}w}{\partial x^{k}}\in {ℒ}_{2}\right\},$$

with the function space of square integrable functions

$${ℒ}_{2}:=\left\{w|\int_{0}^{1}w^{2}\text{d}x<\infty \right\},$$

such that they are twice continuously differentiable in *l*, cf. Reddy (2013). A common method to choose candidates for *φ* and its variation δφ is given by the Bubnov–Galerkin approximation and eventually leads to a system of ODEs.

### 3.2 Bubnov–Galerkin Approximation

In the sense of the Bubnov–Galerkin method, the function *φ* as well as the test function δφ will be approximated by

$$\varphi \left(l,t\right)\approx \varphi^{h}\left(l,t\right)=\sum_{i=1}^{n}a_{i}^{\varphi }\left(t\right)\psi_{i}\left(l\right),$$ (25a)

$$\delta \varphi \left(l,t\right)\approx \delta \varphi^{h}\left(l,t\right)=\sum_{j=1}^{n}b_{j}^{\varphi }\left(t\right)\psi_{j}\left(l\right),$$ (25b)

using the same set of *n* weighted orthogonal spatial basis functions $\psi_{1\ldots n}\in {ℋ}^{2}$, together with *n* time-dependent scaling coefficients $a_{i}^{\varphi }$ for the approximation of the curve tangent angle and $b_{j}^{\varphi }$ for the test function. The weak formulation (Eq. 24) thus reads

$$\begin{array}{l} \frac{1}{c}\sum_{j}b_{j}^{\varphi }\sum_{i}{\left(a_{i}^{\varphi }\right)}_{tt}\int_{0}^{L}\psi_{i}\psi_{j}\text{d}l=\sum_{j}b_{j}^{\varphi }\left[-\sum_{i}a_{i}^{\varphi }\left(\int_{0}^{L}{\left(\psi_{i}\right)}_{ll}{\left(\psi_{j}\right)}_{ll}\text{d}l+{{\left(\psi_{i}\right)}_{lll}\psi_{j}|}_{0}^{L}-{{\left(\psi_{i}\right)}_{ll}{\left(\psi_{j}\right)}_{l}|}_{0}^{L}\right)-\frac{1}{3}\int_{0}^{L}{\left(\sum_{i}a_{i}^{\varphi }{\left(\psi_{i}\right)}_{l}\right)}^{3}{\left(\psi_{j}\right)}_{l}\text{d}l-\frac{1}{3}{\left(\sum_{i}a_{i}^{\varphi }{\left(\psi_{i}\right)}_{l}\right)}^{3}\psi_{j}{|}_{0}^{L}\right]. \end{array}$$ (26)

As the coefficients $b_{j}^{\varphi }$ from the variation Eq. 25b are arbitrary, the weak formulation result in the system of *n* equations

$$\sum_{i}{\left(a_{i}^{\varphi }\right)}_{tt}\int_{0}^{L}\psi_{i}\psi_{j}\text{d}l=c\left[-\sum_{i}a_{i}^{\varphi }\left(\int_{0}^{L}{\left(\psi_{i}\right)}_{ll}{\left(\psi_{j}\right)}_{ll}\text{d}l+{{\left(\psi_{i}\right)}_{lll}\psi_{j}|}_{0}^{L}-{{\left(\psi_{i}\right)}_{ll}{\left(\psi_{j}\right)}_{l}|}_{0}^{L}\right)-\frac{1}{3}(\int_{0}^{L}{\left(\sum_{i}a_{i}^{\varphi }{\left(\psi_{i}\right)}_{l}\right)}^{3}{\left(\psi_{j}\right)}_{l}\text{d}l-{\left(\sum_{i}a_{i}^{\varphi }{\left(\psi_{i}\right)}_{l}\right)}^{3}\psi_{j}{|}^{L}0)\right],$$ (27)

for j=[1,n]. Reorganizing the terms and using a matrix representation finally leads to

$$M{\left(a^{\varphi }\right)}_{tt}=-c\left(Fa^{\varphi }+\frac{1}{3}f^{3}\left(a^{\varphi }\right)\right),$$ (28a)

with the component-wise definitions

$$M_{ij}:=\int_{0}^{L}\psi_{i}\psi_{j}\text{d}l,$$ (28b)

$$F_{ji}:=\int_{0}^{L}{\left(\psi_{i}\right)}_{ll}{\left(\psi_{j}\right)}_{ll}\text{d}l+{{\left(\psi_{i}\right)}_{lll}\psi_{j}|}_{0}^{L}-{{\left(\psi_{i}\right)}_{ll}{\left(\psi_{j}\right)}_{l}|}_{0}^{L},$$ (28c)

$$f_{j}^{3}\left(a^{\varphi }\right):=\int_{0}^{L}{\left(\sum_{i}a_{i}^{\varphi }{\left(\psi_{i}\right)}_{l}\right)}^{3}{\left(\psi_{j}\right)}_{l}\text{d}l-{{\left(\sum_{i}a_{i}^{\varphi }{\left(\psi_{i}\right)}_{l}\right)}^{3}\psi_{j}|}_{0}^{L}.$$ (28d)

Note the index order of the matrix component definitions $M_{ji}$ and $F_{ji}$ that is important for the vector notation in Eq. 28a. In the context of FEM formulations, a particular choice of piecewise orthogonal basis functions ψ is used.

### 3.3 Finite Element Discretization

The core idea of the FEM is to discretize the continuous body into a finite number of *N* elements e∈[1,N], connected at the N+1 node locations $N_{0\ldots N}$, and choose a special set of piecewise function elements $\psi_{1\ldots N}$ that form the global spline approximation $\varphi^{h}\left(l\right)$ of Eq. 25a.

For the weak formulation (Eq. 24), the orthogonal basis functions need to be at least of class ${ℋ}^{2}$, such that the integral-containing second derivatives can be evaluated. In this work, we use, for demonstration purposes, the prominent choice of a Hermite cubic splines with cubic elements $\psi_{1\ldots n}$, constructed by the Hermite local cubic polynomial basis functions:

$$\left[\begin{array}{l} \xi_{1}\left(\lambda \right)\\ \xi_{2}\left(\lambda \right)\\ \xi_{3}\left(\lambda \right)\\ \xi_{4}\left(\lambda \right) \end{array}\right]:=\left[\begin{array}{cccc} 2 & -3 & 0 & 1\\ 1 & -2 & 1 & 0\\ -2 & 3 & 0 & 0\\ 1 & -1 & 0 & 0 \end{array}\right]\left[\begin{array}{l} \lambda^{3}\\ \lambda^{2}\\ \lambda^{1}\\ \lambda^{0} \end{array}\right],\;\lambda \in \left[0,1\right].$$ (29)

**FIGURE 2:**
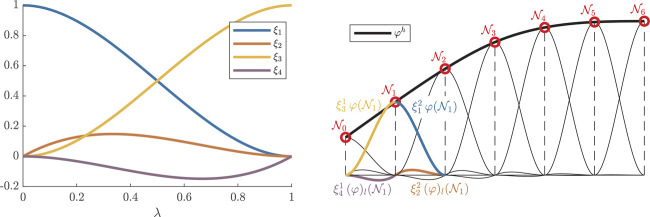
FEM nodes and elements. The local basis functions (Eq. 29) of a unit element are shown on the left. The right plot shows the assembly of N=6 elements to an approximate curve tangent profile $\varphi^{h}$. It further illustrates the role of the local element basis functions $\xi_{1..4}^{e}$ scaled by function values *φ* and ${\left(\varphi \right)}_{l}$ at the node locations $N_{0..6}$.

They have the special property that those coefficients that build the spline element,

$$\psi_{e}\left(\lambda \right):=\underset{=:\xi_{e}^{T}\left(\lambda \right)}{\underset{︸}{\left[\begin{array}{cccc} \xi_{1}^{e}\left(\lambda \right) & \xi_{2}^{e}\left(\lambda \right) & \xi_{3}^{e}\left(\lambda \right) & \xi_{4}^{e}\left(\lambda \right) \end{array}\right]}}\underset{=:a_{e}^{\varphi }}{\underset{︸}{\left[\begin{array}{c} \varphi \left(N_{e-1}\right)\\ {\left(\varphi \right)}_{l}\left(N_{e-1}\right)\\ \varphi \left(N_{e}\right)\\ {\left(\varphi \right)}_{l}\left(N_{e}\right) \end{array}\right]}},$$ (30)

directly correspond to the function value *φ* and its derivative ${\left(\varphi \right)}_{l}$ at the node locations $N_{e-1}$ and $N_{e}$. Figure 2 illustrates the interplay of the local element basis functions and the function values at the node locations to approximate the full curve tangent angle function. Although the full dynamics are encoded in the resulting system of ODEs, what are missing for a well-posed problem are the boundary conditions of a particular simulation case.

### 3.4 Boundary Conditions Expressed in the Curve Tangent Angle

Incorporate the boundary conditions on the curve tangent angle *φ*, and its derivatives follow standard procedures, e.g., Hughes (2012). Because treating position-based boundary conditions is not directly possible in the curve tangent angle beam model (Eq. 21), this chapter focuses on the development of a strategy to express boundary conditions in terms of higher order derivatives only. Without loss of generality, we consider for our beam model a fixed end,

x(l=0,t)=0, (31a)

y(l=0,t)=0, (31b)

and, respectively, its dynamic counterpart

$${\left(x\right)}_{tt}\left(l=0,t\right)=0,$$ (32a)

$${\left(y\right)}_{tt}\left(l=0,t\right)=0.$$ (32b)

Fixing an elastic beam in its position at one end introduces point-wise reaction forces from the mounting onto the beam in *x* and/or *y* directions. While these boundary conditions are straight forward to be incorporated in a FEM formulation for a model in the parameters *x* and *y*, e.g., Eq. 9a, the FEM description of the reduced model (Eq. 28a) directly acts on the tangent angle function *φ* and its derivatives, thus not offering any parameter to incorporate position boundary conditions. However, except for the a-priori known position at the boundary condition itself, the curve tangent angle dynamics Eq. 21 do not require any position parameters to govern the beam profile.

We thus propose to transform the position boundary condition at node $N_{0}$, which cannot be incorporated directly, into a dynamic boundary condition for the neighboring node $N_{1}$ entirely expressed in curve tangent coefficients $a^{\varphi }$. To define this substitutional boundary condition, we first derive another FEM formulation for the beam model parametrized in Cartesian coordinates (Eq. 9a). Not considering external forces for simplicity and recalling the geometric identities Eq. 11, the beam equation Eq. 9a reduces to

$$\left[\begin{array}{c} {\left(x\right)}_{tt}\\ {\left(y\right)}_{tt} \end{array}\right]=c{\left[\begin{array}{c} \sin\left(\varphi \right){\left(\varphi \right)}_{ll}\\ -\cos\left(\varphi \right){\left(\varphi \right)}_{ll} \end{array}\right]}_{l}.$$ (33)

Formulating the weak forms and applying another integration by parts reads

$$\frac{1}{c}\int_{0}^{L}{\left(x\right)}_{tt}\delta x\text{d}l=-\int_{0}^{L}\sin\left(\varphi \right){\left(\varphi \right)}_{ll}{\left(\delta x\right)}_{l}\text{d}l+{\sin\left(\varphi \right){\left(\varphi \right)}_{ll}\delta x|}_{0}^{L},$$ (34a)

$$\frac{1}{c}\int_{0}^{L}{\left(y\right)}_{tt}\delta y\text{d}l=\int_{0}^{L}\cos\left(\varphi \right){\left(\varphi \right)}_{ll}{\left(\delta y\right)}_{l}\text{d}l-{\cos\left(\varphi \right){\left(\varphi \right)}_{ll}\delta y|}_{0}^{L},$$ (34b)

and the Bubnov–Galerkin approximation

$$\begin{array}{cc} x\left(t,l\right)\approx x{\left(t,l\right)}^{h}=\sum_{i=1}^{3}a_{i}^{x}\left(t\right)\psi_{i}\left(l\right), & \delta x\left(t,l\right)\approx \delta x{\left(t,l\right)}^{h}=\sum_{i=1}^{3}b_{i}^{x}\left(t\right)\delta \psi_{i}\left(l\right),\\ y\left(t,l\right)\approx y{\left(t,l\right)}^{h}=\sum_{i=1}^{3}a_{i}^{y}\left(t\right)\psi_{i}\left(l\right), & \delta y\left(t,l\right)\approx \delta y{\left(t,l\right)}^{h}=\sum_{i=1}^{3}b_{i}^{y}\left(t\right)\delta \psi_{i}\left(l\right), \end{array}$$ (35)

with the same set of orthogonal functions ψ that leads to the systems of equations

$$\frac{1}{c}M{\left(a^{x}\right)}_{tt}=f^{x}\left(a^{x}\right),$$ (36a)

$$\frac{1}{c}M{\left(a^{y}\right)}_{tt}=f^{y}\left(a^{y}\right),$$ (36b)

with the components

$$M_{ji}=\int_{0}^{L}\psi_{j}\psi_{i}\text{d}l,$$ (37a)

$$f_{j}^{x}=-\int_{0}^{L}\sin\left(\varphi \right){\left(\varphi \right)}_{ll}{\left(\delta \psi_{j}\right)}_{l}\text{d}l,$$ (37b)

$$f_{j}^{y}=\int_{0}^{L}\cos\left(\varphi \right){\left(\varphi \right)}_{ll}{\left(\delta \psi_{j}\right)}_{l}\text{d}l.$$ (37c)

Note that for both systems of equations in Eq. 36, the matrices M are the same as for the FEM in the curve tangent angle *φ* from Eq. 28a.

Again using Hermite cubic spline basis functions (Eq. 30), the equations of interest in the two systems of *N* equations (Eq. 36) are the ones relating to the position basis function of $N_{1}$, i.e., j=3:

$$\frac{1}{c}\sum_{i}\int_{N_{0}}^{N_{2}}\psi_{3}\psi_{i}\text{d}l{\left(a_{i}^{x}\right)}_{tt}=-\int_{N_{0}}^{N_{2}}\sin\left(\varphi \right){\left(\varphi \right)}_{ll}{\left(\psi_{3}\right)}_{l}\text{d}l,$$ (38a)

$$\frac{1}{c}\sum_{i}\int_{N_{2}}^{N_{2}}\psi_{3}\psi_{i}\text{d}l{\left(a_{i}^{y}\right)}_{tt}=\int_{N_{0}}^{N_{2}}\cos\left(\varphi \right){\left(\varphi \right)}_{ll}{\left(\psi_{3}\right)}_{l}\text{d}l.$$ (38b)

As illustrated in Figure 2, this requires an integration from $N_{0}$ until $N_{2}$ to fully account for all associated local basis functions and thus involves the first six coefficients of the acceleration vectors,

$${\left(a_{1..6}^{x}\right)}_{tt}:=\left[\begin{array}{l} {\left(x\right)}_{tt}\left(N_{0}\right)\\ {\left(x\right)}_{ltt}\left(N_{0}\right)\\ {\left(x\right)}_{tt}\left(N_{1}\right)\\ {\left(x\right)}_{ltt}\left(N_{1}\right)\\ {\left(x\right)}_{tt}\left(N_{2}\right)\\ {\left(x\right)}_{ltt}\left(N_{2}\right) \end{array}\right]\;\text{and}\;{\left(a_{1..6}^{y}\right)}_{tt}:=\left[\begin{array}{l} {\left(y\right)}_{tt}\left(N_{0}\right)\\ {\left(y\right)}_{ltt}\left(N_{0}\right)\\ {\left(y\right)}_{tt}\left(N_{1}\right)\\ {\left(y\right)}_{ltt}\left(N_{1}\right)\\ {\left(y\right)}_{tt}\left(N_{2}\right)\\ {\left(y\right)}_{ltt}\left(N_{2}\right) \end{array}\right],$$ (39)

relating to the function values and first derivatives at the first three nodes. While the right hand side of the FEM formulations (Eq. 36) is already defined in the curve tangent angle *φ*, what remains is to also rewrite coefficient vectors ${\left(a_{1..6}^{x}\right)}_{tt}$ and ${\left(a_{1..6}^{y}\right)}_{tt}$ in terms of *φ* instead of *x* and *y*. Starting again from the geometric identities ${\left(x\right)}_{l}\equiv \cos\left(\varphi \right)$ and ${\left(y\right)}_{l}\equiv \sin\left(\varphi \right)$, the coefficients in Eq. 39 can be expressed as

$${\left(x\right)}_{ltt}\left(l,t\right):=-\sin(\varphi (l,t)){\left(\varphi \left(l,t\right)\right)}_{tt}-\cos\left(\varphi \left(l,t\right)\right){\left(\varphi \left(l,t\right)\right)}_{t}^{2},$$ (40a)

$${\left(x\right)}_{tt}\left(l,t\right):={\left(x\right)}_{tt}\left(0,t\right)-\int_{0}^{l}\sin\left(\varphi \left(s,t\right)\right){\left(\varphi \left(s,t\right)\right)}_{tt}\text{d}s-\int_{0}^{l}\cos\left(\varphi \left(s,t\right)\right){\left(\varphi \left(s,t\right)\right)}_{t}^{2}\text{d}s,$$ (40b)

$${\left(y\right)}_{ltt}\left(l,t\right):=\cos\left(\varphi \left(l,t\right)\right){\left(\varphi \left(s,t\right)\right)}_{tt}\text{d}s-\sin\left(\varphi \left(l,t\right)\right){\left(\varphi \left(l,t\right)\right)}_{t}^{2},$$ (40c)

$${\left(y\right)}_{tt}\left(l,t\right):={\left(y\right)}_{tt}\left(0,t\right)+\int_{0}^{l}\cos\left(\varphi \left(s,t\right)\right){\left(\varphi \left(s,t\right)\right)}_{tt}\text{d}s-\int_{0}^{l}\sin\left(\varphi \left(s,t\right)\right){\left(\varphi \left(s,t\right)\right)}_{t}^{2}\text{d}s,$$ (40d)

containing Volterra integrals with an upper limit *l*. Recalling the spline approximation $\varphi^{h}$ from Eq. 30 and using it for the acceleration terms,

$${\left(\varphi \right)}_{tt}=\sum_{e}{\left(\psi_{e}\right)}_{tt}=\sum_{e}\xi_{e}^{T}{\left(a_{e}^{\varphi }\right)}_{tt},$$ (41)

allows to express the entire FEM balance equations (Eq. 38) in terms of $\varphi^{h}$ with the additional boundary values ${\left(x\right)}_{tt}\left(0,t\right)$ and ${\left(y\right)}_{tt}\left(0,t\right)$ which are, however, known a-priori from the position boundary condition we are incorporating. Eventually, Eq. 38 can be evaluated entirely in *φ* with the left hand sides

$$M_{3,1..6}{\left(a_{3,1..6}^{x}\right)}_{tt}={\underset{=:M_{3,1..6}^{x}}{\underset{︸}{M_{3,1..6}○\left[\begin{array}{c} 0\\ -\sin\left(\varphi \left(N_{0},t\right)\right){\left(\xi_{0}\right)}_{tt}^{T}\left(\lambda =0,t\right)\\ -\int_{0}^{N_{1}}\sin\left(\varphi \left(l,t\right)\right){\left(\xi_{0}\right)}_{tt}^{T}\left(l,t\right)\text{d}s\\ -\sin\left(\varphi \left(N_{1},t\right)\right){\left(\xi_{1}\right)}_{tt}^{T}\left(\lambda =0,t\right)\\ -\int_{0}^{N_{1}}\sin\left(\varphi \left(l,t\right)\right){\left(\xi_{1}\right)}_{tt}^{T}\left(l,t\right)\text{d}l-\int_{N_{1}}^{N_{2}}\sin\left(\varphi \left(l,t\right)\right){\left(\xi_{2}\right)}_{tt}^{T}\left(l-N_{1},t\right)\text{d}l\\ -\sin\left(\varphi \left(N_{2},t\right)\right){\left(\xi_{2}\right)}_{tt}^{T}\left(\lambda =0,t\right) \end{array}\right]}}}^{T}{\left(a_{1..6}^{\varphi }\right)}_{tt},$$ (42a)

and

$$M_{3,1..6}{\left(a_{3,1..6}^{y}\right)}_{tt}=\underset{=:M_{3,1..6}^{y}}{\underset{︸}{M_{3,1..6}○{\left[\begin{array}{c} 0\\ \cos\left(\varphi \left(N_{0},t\right)\right){\left(\xi_{0}\right)}_{tt}^{T}\left(\lambda =0,t\right)\\ \int_{0}^{N_{1}}\cos\left(\varphi \left(l,t\right)\right){\left(\xi_{0}\right)}_{tt}^{T}\left(l,t\right)\text{d}s\\ \cos\left(\varphi \left(N_{1},t\right)\right){\left(\xi_{1}\right)}_{tt}^{T}\left(\lambda =0,t\right)\\ \int_{0}^{N_{1}}\cos\left(\varphi \left(l,t\right)\right){\left(\xi_{1}\right)}_{tt}^{T}\left(l,t\right)\text{d}l\int_{N_{1}}^{N_{2}}\cos\left(\varphi \left(l,t\right)\right){\left(\xi_{2}\right)}_{tt}^{T}\left(l-N_{1},t\right)\text{d}l\\ \cos\left(\varphi \left(N_{2},t\right)\right){\left(\xi_{2}\right)}_{tt}^{T}\left(\lambda =0,t\right) \end{array}\right]}^{T}}}{\left(a_{1..6}^{\varphi }\right)}_{tt},$$ (42b)

where ^○^, denotes an element-wise product, considering the piecewise function element definitions ${\left(\psi_{e}\right)}_{tt}$ from Eq. 30 and the local Hermite cubic base functions $\xi_{e}\left(\lambda ,t\right)$ expressed in the global arc length coordinate $\xi_{e}\left(l,t\right):=\xi_{e}\left(\lambda =l/N,t\right)$. Note that only the first six columns of M affect the position value at $N_{1}$, and all remaining entries of the third row of M are thus zero. The right hand side load value in *x* direction of $N_{1}$ expressed in *φ* reads

$$f_{3}^{x}\left(\varphi \right):=-\int_{N_{0}}^{N_{2}}\sin\left(\varphi \right)+M_{3,1..6}\left[\begin{array}{c} -{\left(x\right)}_{tt}\left(0,t\right)\\ \cos\left(\varphi \left(N_{0},t\right)\right){\left(\varphi \right)}_{t}{\left(N_{0},t\right)}^{2}\\ \int_{0}^{N_{1}}\cos\left(\varphi \left(s,t\right)\right){\left(\varphi \left(s,t\right)\right)}_{t}^{2}\text{d}s\\ \cos\left(\varphi \left(N_{1},t\right)\right){\left(\varphi \right)}_{t}{\left(N_{1},t\right)}^{2}\\ \int_{0}^{N_{2}}\cos\left(\varphi \left(s,t\right)\right){\left(\varphi \left(s,t\right)\right)}_{t}^{2}\text{d}s\\ \cos\left(\varphi \left(N_{2},t\right)\right){\left(\varphi \right)}_{t}{\left(N_{2},t\right)}^{2} \end{array}\right],$$ (42c)

and the load value in *y* direction reads

$$f_{3}^{y}\left(\varphi \right):=\int_{N_{0}}^{N_{2}}\cos\left(\varphi \right)+M_{3,1..6}\left[\begin{array}{c} -{\left(y\right)}_{tt}\left(0,t\right)\\ \sin\left(\varphi \left(N_{0},t\right)\right){\left(\varphi \right)}_{t}{\left(N_{0},t\right)}^{2}\\ \int_{0}^{N_{1}}\sin\left(\varphi \left(s,t\right)\right){\left(\varphi \left(s,t\right)\right)}_{t}^{2}\text{d}s\\ \sin\left(\varphi \left(N_{1},t\right)\right){\left(\varphi \right)}_{t}{\left(N_{1},t\right)}^{2}\\ \int_{0}^{N_{2}}\sin\left(\varphi \left(s,t\right)\right){\left(\varphi \left(s,t\right)\right)}_{t}^{2}\text{d}s\\ \sin\left(\varphi \left(N_{2},t\right)\right){\left(\varphi \right)}_{t}{\left(N_{2},t\right)}^{2} \end{array}\right].$$ (42d)

These expressions are used to incorporate the position boundary conditions into the FEM formulation of the curve tangent angle beam equation.

The resulting system of nonlinear ODEs is used to verify the proposed beam model as well as the presented strategy for incorporating position boundary conditions.

## 4 Simulation Verification

The proposed beam model of Section 2 together with the method for incorporating boundary conditions on lower level derivatives than the descriptive variables of the FEM formulation from Section 3.2 is verified in simulation. First, the developed FEM strategy is tested in two different initial value problems and demonstrated for different magnitudes of external forces and momenta. The second part verifies a dynamic simulation in terms of energy consistency of the beam profile. For both cases, we consider the case of a clamped end at l=0 and a free end at l=L. This is expressed in the following boundary conditions:

x(l=0,t)=0, (43a)

y(l=0,t)=0, (43b)

φ(l=0,t)=0, (43c)

$${\left(\varphi \right)}_{\lambda }\left(l=L,t\right)=0,$$ (43d)

$${\left(\varphi \right)}_{\lambda \lambda }\left(l=L,t\right)=0.$$ (43e)

While the essential boundary condition Eq. 43c and natural boundary conditions Eq. 43d and Eq. 43e are directly considered in the FEM formulation with conventional techniques, conditions Eq. 43a and Eq. 43b are incorporated with the expressions developed in Section 3.4. All simulations have been conducted in MATLAB R2020a.

### 4.1 Initial Value Problem

The static solutions of the FEM formulation (Eq. 28a) with external nodal forces $f^{\text{ext}}$,

$$0=-c\left(Fa^{\varphi }+\frac{1}{3}f^{3}\left(a^{\varphi }\right)+f^{\text{ext}}\right),$$ (44)

are verified in two scenarios. First, the analytic solution of Eq. 17 is replicated with an external moment at the free end, and in the second example, an external nodal force $f^{\text{ext}}$ is applied to the middle of the beam. The initial value problem for both cases is formulated as the nonlinear least-squares problem

$$\underset{a^{\varphi }}{\min}{\left\|\left[\begin{array}{c} -c\left(Fa^{\varphi }+\frac{1}{3}f^{3}\left(a^{\varphi }\right)+f^{\text{ext}}\right)\\ f_{3}^{x}\\ f_{3}^{y} \end{array}\right]\right\|}_{2}^{2},$$ (45)

where the last two rows impose the boundary conditions Eq. 43a and Eq. 43b using the expressions from Eq. 42a and Eq. 42b. The results show that stem from MATLAB’s nonlinear least-squares solver lsqnonlin() for a nominal beam of L=1 m and material parameter c=1, with a FEM discretization into 10 beam elements.

#### 4.1.1 External Moment

**FIGURE 3:**
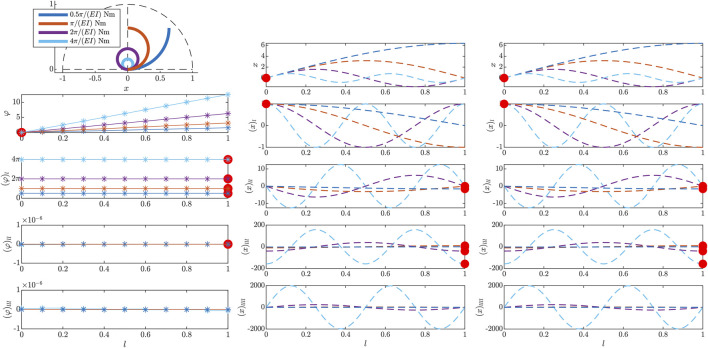
Results of the initial value problem, simulated in the beam tangent angle *φ* for different external momenta at the free end l=L. The results of the actual initial value calculation of *φ* profiles are shown on the left. Additionally, the middle and right columns show the resulting *x* and *y* profiles (dashed lines). The large red dots mark the imposed boundary conditions on the curve tangent angle profile and their respective impact in *x* and *y* direction. Note that the *x* and *y* profiles, as well as the 2D visualization on the top left, are evaluated during postprocessing of the actual simulation results in the curve tangent angle *φ*, applying the geometric identities Eq. 11 and respective derivatives.

Applying an external nodal moment at the free end of the beam results in

$${\left(\varphi \right)}_{ll}=0,$$ (46)

and thus a constant curvature ${\left(\varphi \right)}_{l}$ along the entire beam, according to the analytic solution (Eq. 17). This is also consistent with analytical solutions known in literature Antman (2005). To verify the proposed formulation with this test case, various external momenta proportional to the material parameters mzext⊥(l=L)∈{0.5,1,2,4}π/EI in N m are applied to the free end of the nominal beam. They are incorporated directly as boundary condition (φ)l(l=L)=mzext⊥, substituting Eq. 43d. The results are shown in Figure 3 and depict segments of a perfect circle due to the constant curvature ${\left(\varphi \right)}_{l}$. While ${\left(x\right)}_{l}$ and ${\left(y\right)}_{l}$ components show a FEM approximation of cos(⋅) and sin(⋅) functions, respectively, the according angle *φ* is a purely linear function in this special case and thus can be approximated with arbitrary accuracy, even for a low number of elements. Note that the analytical trigonometric solution is not visually distinguishable from the simulation result and thus is not shown in Figure 3.

#### 4.1.2 External Force

**FIGURE 4:**
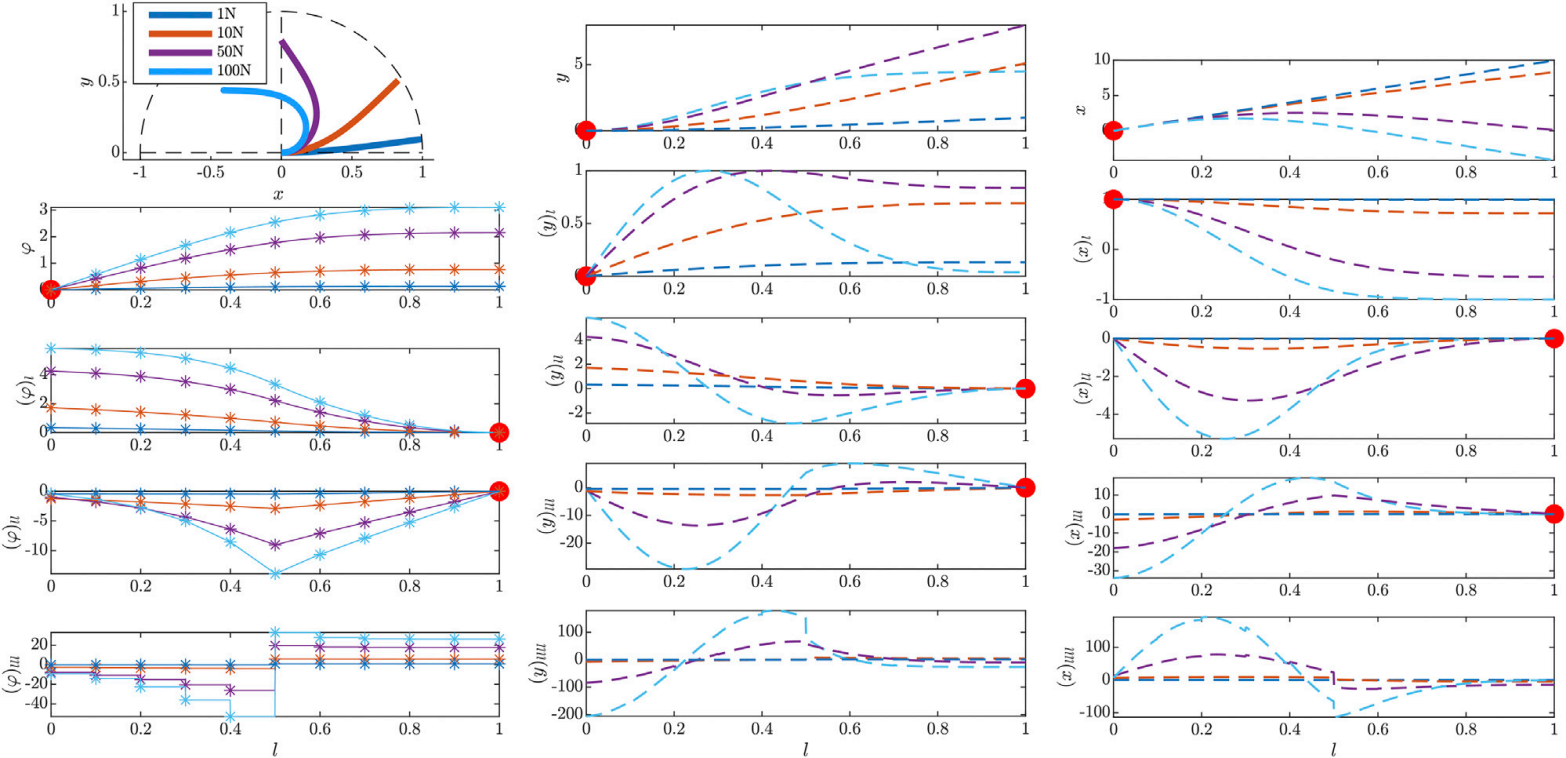
Results of the initial value problem simulated in the beam tangent angle *φ* for different external nodal forces at l=0.5. The results of the actual initial value calculation of *φ* profiles are shown on the left. A detailed explanation of the illustration is given in the caption of Figure 3.

This second example shows the beam deflection under different external nodal forces $f^{\text{ext}}\left(l=\frac{L}{2}\right)\in \left\{1,10,50,100\right\}\;\text{N}$ applied to the middle node of the nominal beam. Physically, this expresses an external force perpendicular to the beam center line, cf. Eq. 20. Results are depicted in Figure 4. While for the two smaller deflections, the beam profiles in *y* direction correspond to results of a common linearized Euler–Bernoulli Beam model, the geometrical nonlinearities show the full effect for the two larger deflection cases. Note that the initial curvature at the clamped end l=0, which is proportional to ${\left(\varphi \right)}_{l}$, is the result of the imposed position boundary condition discussed in Section 3.2. This verifies the effect of the proposed strategy on the equilibrium configuration.

### 4.2 Dynamic Simulation

For the dynamic case, the FEM formulation (Eq. 28a) is again extended by external nodal forces

$$M{\left(a^{\varphi }\right)}_{tt}=-c\left(Fa^{\varphi }+\frac{1}{3}f^{3}\left(a^{\varphi }\right)+f^{\text{ext}}\right),$$ (47)

which allow external perturbation of the system. Assuming that the initial beam configuration φ(l,t=0) complies with the boundary conditions (Eq. 45), the expressions for ${\left(x\right)}_{tt}\left(l=0,t\right)$ and ${\left(y\right)}_{tt}\left(l=0,t\right)$, as developed in Section 3.4, are imposed on the beam, using a Lagrangian multipliers technique. The full system reads

$$\underset{M_{Lag}}{\underset{︸}{\left[\begin{array}{cc} M & {\left(B^{\text{BC}}\right)}^{T}\\ B^{\text{BC}} & 0 \end{array}\right]}}\left[\begin{array}{c} {\left(a^{\varphi }\right)}_{tt}\\ \lambda \end{array}\right]=-c\left[\begin{array}{c} Fa^{\varphi }+\frac{1}{3}f^{3}\left(a^{\varphi }\right)+f^{\text{ext}}\\ g^{\text{BC}} \end{array}\right],$$ (48)

where the position boundary conditions from Eq. 42a are encoded in

$$B^{\text{BC}}:=\left[\begin{array}{cc} M_{3,1..6}^{x} & 0^{T}\\ M_{3.1..6}^{y} & 0^{T} \end{array}\right]\;\text{and}\;g^{\text{BC}}:=\left[\begin{array}{c} f_{3}^{x}\\ f_{3}^{y} \end{array}\right],$$ (49)

with the first six columns of $B^{\text{BC}}$ containing the components from Eqs. 42. The matrix $M_{\text{Lag}}$ is invertible and thus allows solving

$$\left[\begin{array}{c} {\left(a^{\varphi }\right)}_{tt}\\ \lambda \end{array}\right]=-cM_{\text{Lag}}^{-1}\left[\begin{array}{c} Fa^{\varphi }+\frac{1}{3}f^{3}\left(a^{\varphi }\right)+f^{\text{ext}}\\ g^{\text{BC}} \end{array}\right].$$ (50)

The resulting acceleration vector of curve tangent angle coefficients ${\left(a^{\varphi }\right)}_{tt}$ is then integrated to simulate the time-varying trajectories. The results of Figure 4 are computed using MATLAB’s ode45 solver. In a postprocessing step, the energy distribution in the beam is evaluated w.r.t. time *t*.

#### 4.2.1 Energy Analysis

The dynamic simulation result is verified by confirming energy consistency of the simulation result. This is achieved by calculating the total energy,

$$E^{\text{total}}\left(t\right)=E^{\text{pot}}\left(t\right)+E^{\text{kin}}\left(t\right)+E^{\text{ext}}\left(t\right),$$ (51)

consisting of the potential energy $E^{\text{pot}}$, the kinetic energy $E^{\text{kin}}$, and the externally injected energy $E^{\text{ext}}$. While the potential energy is directly proportional to the curvature of the beam center line,

$$E^{\text{pot}}=\frac{1}{2}\int_{0}^{L}EI{\left(\varphi \right)}_{l}^{2}\text{d}l,$$ (52)

It can be directly evaluated from the simulation results. The kinetic energy

$$E^{\text{kin}}=\frac{1}{2}\int_{0}^{L}\rho A\left({\left(x\right)}_{t}^{2}+{\left(y\right)}_{t}^{2}\right)\text{d}l,$$ (53)

however, needs to be rewritten in terms of *φ* first. The geometric identities ${\left(x\right)}_{l}\equiv \text{cos}\left(\varphi \right)$ and ${\left(y\right)}_{l}\equiv \text{sin}\left(\varphi \right)$ together with Schwarz’s theorem on changing the order of derivatives again allow to rewrite the Cartesian components at an arc length *l* of the beam center line, with the Volterra integrals

$${\left(x\right)}_{t}=-\int_{0}^{l}\sin\left(\varphi \left(s,t\right)\right){\left(\varphi \left(s,t\right)\right)}_{t}\text{d}s,$$ (54a)

$${\left(y\right)}_{t}=\int_{0}^{l}\cos\left(\varphi \left(s,t\right)\right){\left(\varphi \left(s,t\right)\right)}_{t}\text{d}s,$$ (54b)

and thus, the kinetic energy of the entire beam can be evaluated with

$$E^{\text{kin}}=\frac{1}{2}\int_{0}^{L}\rho A\left({\left(\int_{0}^{l}\sin\left(\varphi \left(s,t\right)\right){\left(\varphi \left(s,t\right)\right)}_{t}\text{d}s\right)}^{2}+{\left(\int_{0}^{l}\cos\left(\varphi \left(s,t\right)\right){\left(\varphi \left(s,t\right)\right)}_{t}\text{d}s\right)}^{2}\right)\text{d}l.$$ (55)

Note that the rotational component of the kinetic energy is not considered, due to the Euler–Bernoulli assumption of slender beams.

#### 4.2.2 Simulation Results

**FIGURE 5:**
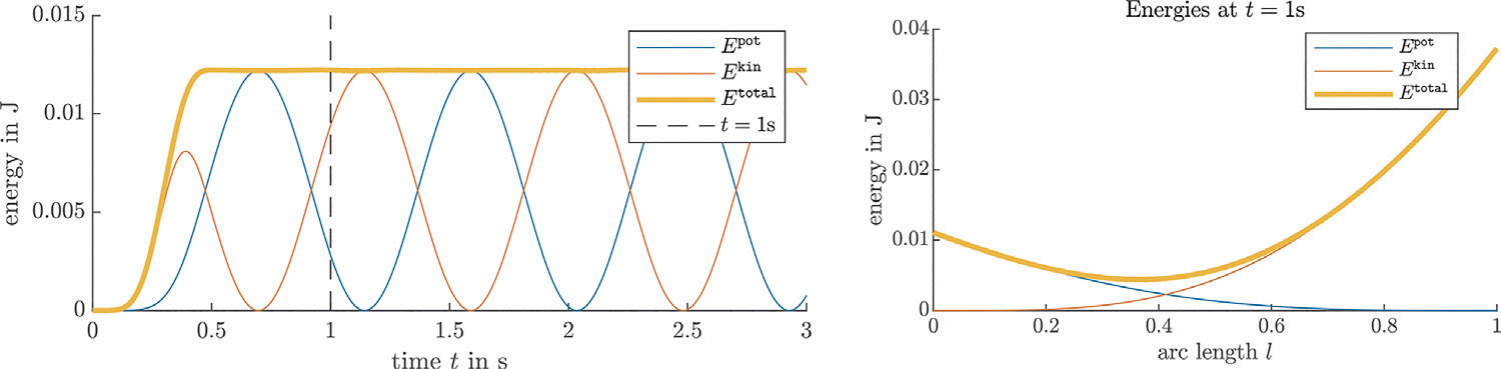
Energy conservation during an FEM simulation of the dynamic curve tangent angle model in a clamped-free scenario, where an external torque is applied to the center of an initially straight beam for 0.5 s. The left plot shows the energy distribution. The right plot shows the energy along the beam at a snapshot taken at *t* = 1 s.

For the dynamic case, an initially unloaded beam with a normalized beam material parameter c=1 and unit length L=1 m is perturbed by an external nodal force,

$$f^{\text{ext}}\left(l=\frac{L}{2},t\right):=\{\begin{array}{cc} f_{\max}^{\text{ext}}\sin{\left(\frac{t}{t_{\max}}\pi \right)}^{2}, & \text{for}\;t<t_{\max},\\ 0, & \text{else,} \end{array}$$ (56)

and again at the beam center, with a maximal unit force $f_{\text{max}}^{\text{ext}}=1$ over a perturbation time of $t_{\text{max}}=0.5\;\text{s}$. The result of the dynamic simulation with 10 beam elements is shown in Figure 5. While in the first 0.5 s, energy is injected in the system via the external perturbation, the resulting beam movement results in an energy exchange between kinetic and potential along the beam while conserving the total amount of energy in the system. Note that no dissipative terms such as damping are considered in the simulated beam (Eq. 21). The right plot of Figure 5 shows the energy distribution within the entire beam at t=1 m. The accumulation of potential energy at the clamped site of the beam again demonstrates the effectiveness of the position boundary condition expressions Section 3.4.

## 5 Conclusion

A model for large planar deformation dynamics of Euler–Bernoulli beams was presented and put into context with well-known more general beam models. Literature does already offer various models that account for arbitrarily large deformations; however, they typically result in a system of coupled nonlinear PDEs expressions. Whereas, the presented approach admits a single-dimensional PDE in one variable, i.e., the curve tangent angle of the beam center line to describe planar beam dynamics under the common Euler–Bernoulli assumptions of shear-free constant cross-sections.

While boundary conditions on the beam profile derivatives—which is sufficient for sliding and/or free ends—can be directly encoded in a simulation algorithm of the curve tangent angle beam model, there is no descriptive variable available to directly incorporate boundary conditions on the beam position—needed for clamped and/or hinged ends. These cases are, however, of course of highly practical relevance. To also address these cases, we additionally outlined a novel method that allows incorporating boundary conditions in FEM formulations, using solely descriptive variables of higher order derivatives. We apply this method to impose position-based boundary conditions in the FEM formulation of the presented beam model, but it is not limited to solely use this case. The strategy is verified in initial value problems, where it replicates analytical solutions and a time-variant FEM simulation by evaluating energy conservation. The presented beam model does not only reduce the computational effort due to the dimensional reduction to a single parameter, but the beam profiles are at the same time less complex in this curve tangent angle parametrization and thus require fewer elements in the FEM description.

Although being nonlinear, the derived beam model provides a concise continuum model for future control theory applications of large deformations, where other more complex model descriptions are not appropriate for current model-based PDE controller development. The model reduction process in this work, however, also outlines more general beam models considering, e.g., shearing, axial torsion, elongation, and/or 3D spatial deformations. This work is thus also a good source for extended beam models including these additional effects, which might be relevant with the advancement of PDE controller development.

## Data Availability

The original contributions presented in the study are included in the article/Supplementary Material, further inquiries can be directed to the corresponding authors.

## Nomenclature

### Beam parameters

*A*: Area of cross-section surface
*E*: Young’s modulus
*G*: Shear modulus
*L*: Total length
ρ: Density
*c*: Constant scaling factor of the PDE $c:=\frac{EI}{\rho A}$

### Vectors

${\hat{g}}_{i}$: Base vector of the coordinate system along the deformed beam i∈[1,3]
${\hat{e}}_{i}$: Base vector of the interial coordinate system with i∈[1,3]
κ: Curvature with respect to ${\hat{g}}_{i}$
*f*: Force vector
*m*: Moment vector
*r*: Vector from the origin to a point on the deformed beam

### Superscripts

${\left(\cdot \right)}^{\text{ext}}$: External quantity
${\left(\cdot \right)}^{\mathrm{inertia}}$: Quantity due to inertia
${\left(\cdot \right)}^{\mathrm{internal}}$: Internal quantity
^║^( ): Parallel component
^┴^( ): Perpendicular component

### Modeling variables

φ: Curve tangent angle between deformed ${\hat{g}}_{1}$ and undeformed beam axis ${\hat{e}}_{1}$
*l*: Beam parameter along beam axis with range [0,L]
*t*: Time
*x*: First component of the location vector along ${\hat{e}}_{1}$
*y*: Second component of the location vector along ${\hat{e}}_{1}$
$E^{\mathrm{def}}$: Potential/kinetic/external/total energy with def∈{pot,kin,ext,total}, respectively
ε: Axial tension parameter $\epsilon :={\left\|{\left(r\right)}_{l}\right\|}_{2}-1$

### Finite element description

*λ*: Local length parameter with range [0,1]
$\psi_{i}$: Global orthogonal basis functions in FEM description
$\xi_{i}$: Hermite local basis functions in FEM description
*N*: Number of elements
$N_{e}$: Node with range e∈[0,N]
*e*: Element number with range [1,N]

### Operators

${\left(\cdot \right)}_{x}$: Partial differentiation of function w.r.t. beam parameter *x*, i.e., $\frac{\partial \left(\cdot \right)}{\partial x}$
${\left(\cdot \right)}_{ijkl}$: Partial differentiation of function w.r.t. parameters i,j,k,l, i.e., $\frac{\partial^{4}\left(\cdot \right)}{\partial i\partial j\partial k\partial l}$
δ(⋅): Variation of function, e.g., δφ
${\|(\cdot )\|}_{2}$: Euclidean norm $\sqrt{\sum_{i}{\left(\cdot \right)}_{i}^{2}}$
(⋅)∘(⋅): Hadamard or element-wise product.

## References

[B1] Antman S. S. (2005). Planar steady-state problems for elastic rods. Nonlinear problems of elasticity. 2 edn. New York, United States: Springer Science & Business Media Chap. 4, 95–134.

[B2] Bernoulli J. (1691). Quadratura curvae, e cujus evolutione describitur inflexae laminae curvatura. Die Werke von Jakob Bernoulli 5, 223–227.

[B3] Bernoulli D. (1742). The 26th letter to euler. Correspondence Mathématique et Physique 2, 499.

[B4] Bernoulli J. (1694). Curvatura laminae elasticae. Acta Eruditorum Lipsiae 1694, 262–276.

[B5] Bernstein B. Hall D. A. Trent H. M. (1958). On the dynamics of a bull whip. The J. Acoust. Soc. America 30, 1112–1115. 10.1121/1.1909473

[B6] Bicchi A. Tonietti G. (2004). Fast and “Soft-Arm” tactics. IEEE Robot. Automat. Mag. 11, 22–33. 10.1109/mra.2004.1310939

[B7] Bresse J. A. C. (1859). Cours de mecanique appliquee: Re’sistance des mate’riaux et stabilite’des constructions, (Paris, France: Mallet-Bachelier). 1.

[B8] Bretl T. McCarthy Z. (2014). Quasi-static manipulation of a Kirchhoff elastic rod based on a geometric analysis of equilibrium configurations. Int. J. Robotics Res. 33, 48–68. 10.1177/0278364912473169

[B9] Crisfield M. A. Jelenić G. (1999). Objectivity of strain measures in the geometrically exact three-dimensional beam theory and its finite-element implementation. Proc. R. Soc. Lond. A. 455, 1125–1147. 10.1098/rspa.1999.0352

[B10] Duriez C. (2013). Control of elastic soft robots based on real-time finite element method. In International Conference on Robotics and Automation (ICRA) (IEEE), 3982–3987. 10.1109/ICRA.2013.6631138

[B11] Elishakoff I. (2020). Who developed the so-called timoshenko beam theory? Math. Mech. Sol. 25, 97–116. 10.1177/1081286519856931

[B12] Euler L. (1744). Methodus inveniendi lineas curvas maximi minimive proprietate gaudentes sive solutio problematis isoperimetrici latissimo sensu accepti. Genevae, Switzerland: Marcus-Michael Bousquet), 1.

[B13] Gurtin M. E. (1982). An introduction to continuum mechanics. Cambridge, MA: Academic Press.

[B14] Haddadin S. Krieger K. Albu-Schäffer A. (2011). Exploiting elastic energy storage for cyclic manipulation: modeling, stability, and observations for dribbling. (CDC-ECC) (IEEE), 690–697. 10.1109/CDC.2011.6161022

[B15] Haddadin S. Laue T. Frese U. Wolf S. Albu-Schäffer A. Hirzinger G. (2009). Kick it with elasticity: safety and performance in human-robot soccer. Robotics Autonomous Syst. 57, 761–775. 10.1016/j.robot.2009.03.004

[B16] Hegarty G. Taylor S. (2012). Classical solutions of nonlinear beam equations: existence and stabilization. SIAM J. Control. Optim. 50, 703–719. 10.1137/100793864

[B17] Hughes T. J. (2012). The finite element method: linear static and dynamic finite element analysis. (Chelmsford, MA: Courier Corporation).

[B18] Ito K. (2001). Numerical study for the stability of a geometrically nonlinear elastic beam with velocity feedback. Nonlinear Anal. Theor. Methods Appl. 47, 3813–3821. 10.1016/s0362-546x(01)00500-4

[B19] Kirchhoff G. (1859). Ueber das gleichgewicht und die bewegung eines unendlich dünnen elastischen stabes. J. für die reine Angew. Mathematik 1859, 285–313.

[B20] Krstic M. Siranosian A. A. Smyshlyaev A. (2006a). Backstepping boundary controllers and observers for the slender timoshenko beam: Part Ⅰ-design. American control conference. (ACC) (IEEE), Minneapolis, MN, 24 July, 2006, 2412–2417. 10.1109/ACC.2006.1656581

[B21] Krstic M. Siranosian A. A. Smyshlyaev A. Bement M. (2006b). Backstepping boundary controllers and observers for the slender timoshenko beam: Part Ⅱ—stability and simulations. Conference on Decision and Control (CDC) (IEEE), San Diego, CA, 13-15 December, 2006, 3938–3943. 10.1109/CDC.2006.377717

[B22] Levien R. (2008). The elastica: a mathematical history. Berkeley, CA: University of California. Technical Report No. UCB/EECS-2008-103.

[B23] Love A. E. H. (1892). A treatise on the mathematical theory of elasticity. Cambridge, United Kingdom: The University Press.

[B24] Malzahn J. Schloss R. Bertram T. (2015). Link elasticity exploited for payload estimation and force control. International Conference on Intelligent Robots and Systems (IROS) (IEEE), Hamburg, Germany, 28 September–2 October, 2015, 1508–1513. 10.1109/IROS.2015.7353567

[B25] McCarragher B. J. (2000). “A hybrid position/force approach to the exploitation of elasticity in manipulation,” in Robot manipulation of deformable objects. Editors Henrich D. Wörn H. (London, United Kingdom: Springer London), 91–109. 10.1007/978-1-4471-0749-1_7

[B26] McMillen T. Goriely A. (2003). Whip waves. Physica D: Nonlinear Phenomena 184, 192–225. 10.1016/s0167-2789(03)00221-5

[B27] Meier C. Popp A. Wall W. A. (2015). A locking-free finite element formulation and reduced models for geometrically exact Kirchhoff rods. Comp. Methods Appl. Mech. Eng. 290, 314–341. 10.1016/j.cma.2015.02.029

[B28] Meier C. Popp A. Wall W. A. (2019). Geometrically exact finite element formulations for slender beams: Kirchhoff-Love theory versus simo-reissner theory. Arch. Computat Methods Eng. 26, 163–243. 10.1007/s11831-017-9232-5

[B29] Meirovitch L. Baruh H. (1983). On the problem of observation spillover in self-adjoint distributed-parameter systems. J. Optim Theor. Appl 39, 269–291. 10.1007/bf00934533

[B30] Padhi R. Ali S. F. (2009). An account of chronological developments in control of distributed parameter systems. Annu. Rev. Control. 33, 59–68. 10.1016/j.arcontrol.2009.01.003

[B31] Pekarovskiy A. Saluja K. Sarkar R. Buss M. (2014). Resonance-driven dynamic manipulation: dribbling and juggling with elastic beam. International conference on robotics and automation (ICRA) (IEEE), Hong Kong, China, 31 May–7 June 2014, 943–948. 10.1109/ICRA.2014.6906967

[B32] Pham H. Pham Q.-C. (2017). Robotic manipulation of a rotating chain. IEEE Trans. Robotics 34, 139–150. 10.1109/TRD.2017.2775650

[B33] Rayleigh J. W. S. B. (1877). The theory of sound, 1. New York, NY: Macmillan.

[B34] Reddy B. D. (2013). Introductory functional analysis: with applications to boundary value problems and finite elements, 27. New York, NY, United States: Springer Science & Business Media.

[B35] Reissner E. (1981). On finite deformations of space-curved beams. Z. Angew. Math. Phys. 32, 734–744. 10.1007/bf00946983

[B36] Reissner E. (1972). On one-dimensional finite-strain beam theory: the plane problem. J. Appl. Math. Phys. (Zamp) 23, 795–804. 10.1007/bf01602645

[B37] Romero I. Urrecha M. Cyron C. J. (2014). A torsion-free non-linear beam model. Int. J. Non-Linear Mech. 58, 1–10. 10.1016/j.ijnonlinmec.2013.08.008

[B38] Simo J. C. (1985). A finite strain beam formulation. The three-dimensional dynamic problem. Part I. Comp. Methods Appl. Mech. Eng. 49, 55–70. 10.1016/0045-7825(85)90050-7

[B39] Spolek G. A. (1986). The mechanics of flycasting: the flyline. Am. J. Phys. 54, 832–836. 10.1119/1.14425

[B40] Tantanawat T. Kota S. (2007). Design of compliant mechanisms for minimizing input power in dynamic applications. J. Mech. Des. 129, 1064–1075. 10.1115/1.2756086

[B41] Tavasoli A. (2015). Dynamic modeling and nonlinear boundary control of hybrid Euler-Bernoulli beam system with a tip mass. Proc. IMechE 229, 3–15. 10.1177/1464419314541790

[B42] Timoshenko S. P. (1916). A course of elasticity theory. Part 2: rods and plates. St. Petersburg, Russia: A.E. Collins Publishers.

[B43] Timoshenko S. P. (1921). LXVI. On the correction for shear of the differential equation for transverse vibrations of prismatic bars. Lond. Edinb. Dublin Philos. Mag. J. Sci. 41, 744–746. 10.1080/14786442108636264

